# The Diagnostic Utility of Anti-cyclic Citrullinated Peptide Antibodies, Matrix Metalloproteinase-3, Rheumatoid Factor, Erythrocyte Sedimentation Rate, and C-reactive Protein in Patients with Erosive and Non-erosive Rheumatoid Arthritis

**DOI:** 10.1080/17402520500233510

**Published:** 2005-09

**Authors:** O. Shovman, B. Gilburd, G. Zandman-Goddard, Y. Sherer, H. Orbach, R. Gerli, Y. Shoenfeld

**Affiliations:** 1 Center for Autoimmune Diseases Department of Medicine ‘B’ Sheba Medical Center Israel; 2 Department of Medicine ‘B’ Wolfson Medical Center Sackler Faculty of Medicine Tel-Aviv University Holon Israel; 3 Dipartmrnt Di Medicina Clinica e Sperimentale Universita Di Perugia Sezione di Medicina Interna e Scienze Oncologiche Policlinico Monteluco Perugia Italy; 4 Incumbent of the Laura Schwarz-Kipp Chair for Research of Autoimmune Diseases Tel-Aviv University Tel-Hashomer Israel

## Abstract

*Objective*: To compare the diagnostic utility of laboratory variables, including matrix metalloproteinase-3 (MMP-3), anti-cyclic citrullinated peptide (CCP) antibodies, rheumatoid factor (RF), erythrocyte sedimentation rate (ESR), and C-reactive protein (CRP) in patients with erosive and non-erosive rheumatoid arthritis (RA).

*Methods*: We assembled a training set, consisting of 60 patients with RA, all fulfilling the revised criteria of the American College of Rheumatology. A commercial enzyme linked immunosorbent assay (ELISA) was used both to test for anti-CCP antibodies (second generation ELISA kit) and MMP; RF were detected by latex-enhanced immunonephelometric assay. CRP was measured by latex turbidimetric immunoassay.

*Results*: The levels of anti-CCP antibody titers and ESR were significantly higher in patients with erosive disease than those in non-erosive RA patients (*p* < 0.001 and 0.0341) respectively. Moreover, a higher frequency of elevated titers of anti-CCP antibodies was found in RA patients with erosions compared to patients with non-erosive RA (78.3% vs. 43.2% respectively). The ROC curves of anti-CCP passed closer to the upper left corner than those other markers and area under the curve (AUC) of anti-CCP was significantly larger than AUC of other markers (0.755 for anti-CCP, 0.660 for ESR, 0.611 for CRP, 0.577 for RF, and 0.484 for MMP-3 female).

A positive predictive value was higher for anti-CCP antibodies in comparison to other markers. We did not find significant statistical correlation between anti-CCP antibody titers and inflammatory markers such as ESR or CRP. However, we confirmed the correlation of elevated titers of anti-CCP antibodies and RF in both groups of patients whereas the degree of correlation was more significant in non-erosive patients.

*Conclusion*: The results of our study suggest that the presence of elevated anti-CCP antibody titers have better diagnostic performance than MMP-3, RF, CRP and ESR in patients with erosive RA.


*Clinical & Developmental Immunology*, September 2005; 12(3): 197–202

Taylor & Francis

Taylor & Francis Group

### The diagnostic utility of anti-cyclic citrullinated peptide antibodies, matrix metalloproteinase-3, rheumatoid factor, erythrocyte sedimentation rate, and C-reactive protein in patients with erosive and non-erosive rheumatoid arthritis

O. SHOVMAN[1], B. GILBURD[1], G. ZANDMAN-GODDARD[1], Y. SHERER[1], H. ORBACH[2], R. GERLI[3], & Y. SHOENFELD[1,4]

[1]*Center for Autoimmune Diseases, Department of Medicine 'B', Sheba Medical Center, Israel,* [2]*Department of Medicine 'B' Wolfson Medical Center, Holon, Israel; Sackler Faculty of Medicine, Tel-Aviv University, Israel,* [3]*Dipartmrnt Di Medicina Clinica e Sperimentale, Universita Di Perugia, Sezione di Medicina Interna e Scienze Oncologiche Policlinico Monteluco, Perugia, Italy, and* [4]*Incumbent of the Laura Schwarz-Kipp Chair for Research of Autoimmune Diseases, Tel-Aviv University, Tel-Hashomer, Israel*

**Abstract**

*Objective:* To compare the diagnostic utility of laboratory variables, including matrix metalloproteinase-3 (MMP-3), anti-cyclic citrullinated peptide (CCP) antibodies, rheumatoid factor (RF), erythrocyte sedimentation rate (ESR), and C-reactive protein (CRP) in patients with erosive and non-erosive rheumatoid arthritis (RA).

*Methods:* We assembled a training set, consisting of 60 patients with RA, all fulfilling the revised criteria of the American College of Rheumatology. A commercial enzyme linked immunosorbent assay (ELISA) was used both to test for anti-CCP antibodies (second generation ELISA kit) and MMP; RF were detected by latex-enhanced immunonephelometric assay. CRP was measured by latex turbidimetric immunoassay.

*Results:* The levels of anti-CCP antibody titers and ESR were significantly higher in patients with erosive disease than those in non-erosive RA patients ($p < 0.001$ and 0.0341) respectively. Moreover, a higher frequency of elevated titers of anti-CCP antibodies was found in RA patients with erosions compared to patients with non-erosive RA (78.3% vs. 43.2% respectively). The ROC curves of anti-CCP passed closer to the upper left corner than those other markers and area under the curve (AUC) of anti-CCP was significantly larger than AUC of other markers (0.755 for anti-CCP, 0.660 for ESR, 0.611 for CRP, 0.577 for RF, and 0.484 for MMP-3 female).

A positive predictive value was higher for anti-CCP antibodies in comparison to other markers. We did not find significant statistical correlation between anti-CCP antibody titers and inflammatory markers such as ESR or CRP. However, we confirmed the correlation of elevated titers of anti-CCP antibodies and RF in both groups of patients whereas the degree of correlation was more significant in non-erosive patients.

*Conclusion:* The results of our study suggest that the presence of elevated anti-CCP antibody titers have better diagnostic performance than MMP-3, RF, CRP and ESR in patients with erosive RA.

**Keywords:** *Rheumatoid factor (RF), anti-CCP antibodies, C-reactive protein (CRP), erythrocyte sedimentation rate (ESR), rheumatoid arthritis (RA)*

### Introduction

Rheumatoid arthritis (RA) is a systemic autoimmune disease of unknown etiology, distinguished by chronic inflammation of joints resulting in tissue degradation and joint deformation. The course of RA is varied ranging from a mild to an aggressive form. Early diagnosis and treatment reduce joint destruction, preserve function and improve survival (Subcommittee 2002).

The link between chronic inflammation and joint damage has been widely established, especially the relevance of inflammatory markers such as erythrocyte

Correspondence: Y. Shoenfeld, Department of Medicine 'B' & Center for Autoimmune Diseases, Chaim Sheba Medical Center, Tel-Hashomer 52621, Israel. Tel: 972 3 5302652. Fax: 972 3 5352855. E-mail: shoenfel@post.tau.ac.il

ISSN 1740-2522 print/ISSN 1740-2530 online © 2005 Taylor & Francis
DOI: 10.1080/17402520500233510

sedimentation rate (ESR) and C reactive protein (CRP) (Graudal et al. 2000, Plant et al. 200l). However, the damage may progress in spite of decreased inflammatory activity and erosions may develop in patients without clinical sings of significant inflammation (Kirwan 1997, van den Berg 2001). Therefore, an identification of reliable predictors and markers of joint damage is necessary.

In the present study, we selected several laboratory variables and tested their prognostic value in a well defined cohort of patients with erosive and non-erosive RA. The variables were ESR and CRP, reflecting inflammation; matrix metalloproteinase-3 (MMP-3), which is involved in matrix degradation and cartilage turnover and a set of autoantibodies: rheumatiod factor (RF) and anti-cyclic citrullinated peptide antibody (anti-CCP).

MMP-3 plays an important role in the pathogenesis of matrix degradation in RA, including proteoglycans, gelatins, laminin, fibronectin and collagen (Okada et al. 1987). An over-expression of MMP-3 in synovial fluid, rheumatoid synovium and cartilage as well as an increased level of MMP-3 in the serum obtained from RA patients clearly reflects the contribution of MMP-3 in chronic inflammation and joint destruction (Manicourt et al. 1995, Yoshihara et al. 1999). In addition, serum MMP-3 levels correlate with clinical activity of RA (Keyszer et al. 1999, So et al. 1999). The data regarding the relevance of serum MMP-3 levels and the presence of joint erosions remains controversial (So et al. 1999, Posthumus et al. 2000).

RA is associated with elevated titers of antibodies, including RF, anti-CCP, antibodies directed against RA-33, calpastatin, keratin and antifilaggrin; most of these have failed to demonstrate adequate diagnostic and prognostic value (Goldbach-Mansky et al. 2000). RF, an autoantibody directed against the constant region of IgG is elevated in 75% of patients with RA and widely used in clinical practice. In addition to RF, anti-CCP antibodies are frequently observed in patients with RA, especially in early disease (Ranta-paa-Dahlqvist et al. 2003, Nielen et al. 2004). It has been reported that elevated titers of anti-CCP antibodies are more specific for RA than RF with a disease specificity approaching 100% (Schellekens et al. 2000). Both of these serological markers are associated with more severe joint damage (Visser et al. 2002, Orbach et al. 2002, Vencovsky et al. 2003, Meyer et al. 2003, Forslind et al. 2004). The comparison of diagnostic utility of anti-CCP, RF and MMP-3 in patients with RA and other autoimmune diseases suggest that anti-CCP proved to be superior to RF and MMP-3 (Suzuki et al. 2003). A high disease specificity of anti-CCP coupled with reasonable sensitivity and high predictive value for RA progression and radiological damage suggest that anti-CCP may play an important role in RA pathogenesis.

The objective of this study was to compare the diagnostic utility of laboratory variables, including MMP-3, anti-CCP antibodies, RF, ESR and CRP in 60 samples, collected from patients with erosive and non-erosive RA.

### Patients and methods

### *Patients*

Sixty patients with RA, all fulfilling the revised criteria of the American College of Rheumatology were included in the study. Serum samples were obtained from Dr R. Gerli (Universita Di Perugia, Italy). The patients were divided into two groups: erosive and non-erosive disease according to the presence of erosions on X-ray.

The median disease duration of RA was 5–10 years. Twenty three patients (15 female and 8 male) had erosive disease and 37 (29 female and 8 male) had non-erosive disease. The median age of the patients was 62 and 60 years in the first and second group, respectively.

All patients' sera were tested for anti-CCP, RF, MMP-3, CRP, and ESR.

### *Methods*

Anti-CCP antibody titers was detected using a commercial Quanta Lite CCP ELISA anti-CCP 2 kit (INOVA Diagnostics, San Diego, CA, USA). The optimal cut-off value for anti-CCP ELISA was 20 U/ml.

RF was measured by latex-enhanced immuno-nephelometric assay (Dede Behring, Marburg, Germany). The cut-off value for RF was 15 IU/ml. MMP-3 was measured by ELISA (The Binding Site Limited, Birmingham, UK). The cut-off values for MMP-3 were 45.3 ng/ml for males and 21.0 ng/ml for females. CRP was measured by latex turbiimetric immunoassay (Medical and Biological Laboratories, Nagoya, Japan). ESR was measured by Westergren method.

### *Statistical analysis*

Comparison of the level distributions of anti-CCP, RF, MMP-3, ESR and CRP in patients with erosive and non-erosive disease was made using the Mann Whitney *U* test. Differences between groups of patients were considered significant when $P$ values were $< 0.05$. Comparisons of sensitivity and specificity were made using McNemar's test.

For the construction of ROC curves, relations between sensitivity (ordinate) and specificity (abscissa) for various cut-off points were plotted. In general, a closer location of the ROC plot to the upper left corner indicates a higher diagnostic performance

Table I. Demographic and laboratory characteristics of patients with erosive and non-erosive arthritis.

| | Erosive RA ($n$=23) | Non-erosive RA ($n$=37) |
|---|---|---|
| Age | 62.15 (11.28) | 60 (12.26) |
| Sex female | 15 | 29 |
| Sex male | 8 | 8 |
| MMP3 Male (ng/ml) | 23.26(14.3) | 40.33(40.2) |
| MMP3 Female (ng/ml) | 19.29(22) | 17.19(30.89) |
| ESR (mm/h) | 35.65(25.88) | 22.49(15.11) |
| RF (IU/ml) | 1.17(0.94) | 0.89(0.97) |
| Anti-CCP (U/ml) | 109.58(61.07) | 53.25(60.75) |
| CRP (mg/l) | 1.99(3.21) | 1.65(2.38) |

The results are shown as mean (STDEV); MMP-3, matrix metalloproteinase 3; ESR, erythrocyte sedimentation rate; RF, rheumatoid factor; Anti-CCP, antibodies against cyclic citrullinated peptide; CRP, C reactive protein.

of the assay. The area under the ROC curve (AUC) provides an index of the overall discriminative ability of the test. The comparison of AUC was performed utilizing the Statistical Package SPSS. Pearson's correlation coefficient assessed the importance of the different variables. Differences were considered significant if $p < 0.05$. The determination of the predictive value was done by MedCalc Software.

### Results

*Serum levels of anti-CCP, MMP-3, RF, CRP and ESR in RA patients with erosive and non-erosive disease*

We examined the levels of anti-CCP, MMP-3, RF, CRP and ESR level in RA patients with erosive and non-erosive disease (Table I). The levels of anti-CCP antibody titers and ESR were significantly higher in patients with erosive disease than those with non-erosive disease ($p <$ 0.001 and 0.0341 respectively).

Moreover, the higher frequency of elevated anti-CCP antibody titers was found in RA patients with erosions compared to the value in patients with non-erosive RA (78.3% vs. 43.2%) (Table II).

*Clinical sensitivity and specificity of anti-CCP, MMP-3, RF, CRP and ESR*

Elevated titers of anti-CCP antibodies had a specificity and sensitivity of 70.3% and 73.9%, respectively for erosive RA compared to non-erosive disease (Table III). This clinical specificity of anti-CCP antibodies was superior to RF, ESR, CRP and MMP (Table III). For further comparison of the diagnostic utilities of each tests, we constructed ROC curves and calculated the AUC. The ROC curves of anti-CCP passed closer to the upper left corner than the other markers indicating that the sensitivity compared at the same specificity value, was higher for anti-CCP antibody titers (Figure 1). The superiority of anti- CCP to other markers was confirmed by comparing AUC, since AUC of anti-CCP was significantly larger than AUC of other markers (area under the curve was 0.755 for anti-CCP, 0.660 for ESR, 0.611 for CRP, 0.577 for RF, and 0.484 for MMP-3 female). Therefore, it appears that anti-CCP has a higher diagnostic performance for diagnosis of erosive RA.

Table II. Frequency of positive results of MMP3, ESR, RF, anti-CCP and CRP in patients with erosive and non-erosive RA.

| Variable (%) | Non-erosive RA | Erosive RA |
|---|---|---|
| MMP3 female | 24.1 | 40 |
| MMP3 male | 37.5 | 0 |
| ESR | 32.4 | 56.5 |
| RF | 45 | 57 |
| Anti-CCP | 43.2 | 78.3 |
| CRP | 21.6 | 26.1 |

MMP-3, matrix metalloproteinase 3; ESR, erythrocyte sedimentation rate; RF, rheumatoid factor; Anti-CCP, antibodies against cyclic citrullinated peptide; CRP, C reactive protein.

*Predictive value of anti-CCP, MMP-3, RF, CRP and ESR*

The positive predictive value was higher for anti-CCP (60.7%) and MMP3 in males (61.5%). The negative predictive value was higher for MMP3 in males (100%), CRP (87.5%) and anti-CCP antibody titers (81.2%). To note, that the high positive and negative predictive value for MMP 3 was received from a very small group (8 patients) and can not be statistically significant. Thus, anti-CCP is the best predictor for erosive disease compared to the other markers (Table IV).

Table III. Determination of sensitivity and specificity of MMP3, ESR, RF, anti-CCP and CRP in patients with erosive and non-erosive RA.

| Variables | Criterion | Sensitivity (95% C.I.) | Specificity (95% C.I.) |
|---|---|---|---|
| MMP3 Female (ng/ml) | 22.570 | 33.3 (11.9–61.6) | 86.2 (68.3–96.0) |
| MMP3 Male (ng/ml) | 49.260 | 100.0 (100.0–100.0) | 37.5 (9.0–75.3) |
| ESR (mm/hr) | 21.000 | 69.6 (47.1–86.7) | 56.8 (39.5–72.9) |
| RF (IU/ml) | 0.000 | 65.2 (42.7–83.6) | 51.4 (34.4–68.1) |
| Anti-CCP (U/ml) | 88.360 | 73.9 (51.6–89.7) | 70.3 (53.0–84.1) |
| CRP (mg/l) | 0.400 | 91.3 (71.9–98.7) | 37.8 (22.5–55.2) |

MMP-3, matrix metalloproteinase 3; ESR, erythrocyte sedimentation rate; RF, rheumatoid factor; Anti-CCP, antibodies against cyclic citrullinated peptide; CRP, C reactive protein.

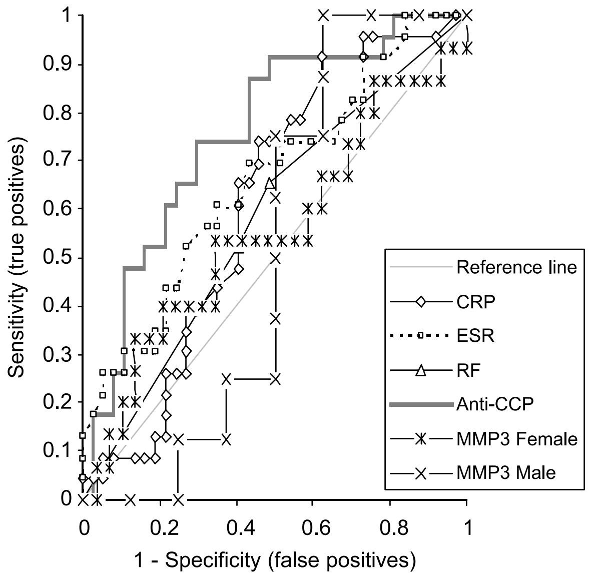

Figure 1. ROC curves of anti-CCP, MMP3 (male and female), RF, ESR and CRP.

*Correlation of anti-CCP and RF levels with inflammatory markers in erosive and non-erosive RA*

As shown in Tables V and VI, we did not find a correlation between elevated levels of anti-CCP antibodies and inflammatory markers, including ESR and CRP in the two groups of RA patients.

In contrast, there was a correlation between levels of RF and CRP in non-erosive RA. A more significant correlation was seen between elevated anti-CCP antibody titers and RF in patients with non-erosive RA ($p < 0.001$) compared to that in patients with erosive RA ($p < 0.046$).

### Discussion

Joint damage accounts for a considerable part of disability caused by RA. Early diagnosis and prevention of joint damage is an important gold standard of treatment. Hence, an identification of the reliable disease predictors may modify the disease course.

In the present study, we compared the diagnostic utility of anti-CCP antibodies and other laboratory markers such as MMP-3, RF IgM, ESR and CRP in patients with erosive and non-erosive RA. We found that the levels and the frequency of elevated titers of anti-CCP antibodies and ESR were higher in patients with erosive RA. Based on ROC curves analyses, we demonstrated that the presence of anti-CCP antibodies in patients with erosive RA has a better diagnostic performance than MMP-3, RF, CRP and ESR. A positive predictive value was higher for anti-CCP antibodies in comparison to others markers. We did not find significant statistical correlation of anti-CCP antibodies with inflammatory markers such as ESR and CRP in patients with erosive RA as well as in patients without erosions. At the same time, we confirmed a correlation of anti-CCP and RF in both groups of patients whereas, the degree of correlation was stronger in non-erosive patients. Thus, the diagnostic utility of anti-CCP antibodies was superior to other markers for erosive RA. Furthermore, absence of correlation between anti-CCP levels and inflammatory markers in patients with erosive disease came out as an important self-determining marker of erosions.

In recent years interesting data have been accumulated regarding the diagnostic utility of anti-CCP antibodies in RA patients and their role in the pathogenesis. Most of clinical utility of this test is associated with high disease specificity (Schellekens et al. 2000) and the presence of anti-CCP antibodies in early phases of RA (Rantapaa-Dahlqvist et al. 2003). Moreover it has been demonstrated by Nielen et al. (2004) that the appearance of anti-CCP antibodies in the circulation may occur several years before the RA onset and represent a marker of future disease.

Table IV. Detection of positive and negative predictive values of MMP3, ESR, RF, Anti-CCP antibodies and CRP in patients with erosive and non-erosive RA.

| Variables | Criterion | "+PV" | " − PV" |
|---|---|---|---|
| MMP3 Female (ng/ml) | 22.57 | 55.6 | 71.4 |
| MMP3 Male (ng/ml) | 49.26 | 61.5 | 100.0 |
| CRP (mg/l) | 0.40 | 47.7 | 87.5 |
| ESR (mm/hr) | 21.00 | 50.0 | 75.0 |
| RF (IU/ml) | 0.00 | 45.5 | 70.4 |
| Anti-CCP (U/ml) | 88.36 | 60.7 | 81.2 |

MMP-3, matrix metalloproteinase 3; ESR, erythrocyte sedimentation rate; RF, rheumatoid factor; Anti-CCP, antibodies against cyclic citrullinated peptide; CRP, C reactive protein; +PV—positive predictive value; −PV—negative predictive value.

Table V. Pearson correlation coefficients between ESR, RF, anti-CCP and CRP in non-erosive patients.

| | Anti-CCP | | CRP | | ESR | | RF | |
|---|---|---|---|---|---|---|---|---|
| | *r* | *p*-value | *r* | *p*-value | *r* | *p*-value | *r* | *p*-value |
| Anti-CCP | × | × | −0.16760 | 0.3215 | 0.06579 | 0.6989 | 0.66310 | < 0.0001 |
| CRP | −0.16760 | 0.3215 | × | × | 0.09177 | 0.5890 | 0.34820 | 0.0347 |
| ESR | 0.06579 | 0.6989 | 0.09177 | 0.5890 | × | × | 0.12550 | 0.4592 |
| RF | 0.66310 | < 0.0001 | 0.34820 | 0.0347 | 0.12550 | 0.4592 | × | × |

Table VI. Pearson correlation coefficients between ESR, RF, anti-CCP and CRP in erosive patients.

| | Anti-CCP | | CRP | | ESR | | RF | |
|---|---|---|---|---|---|---|---|---|
| | *r* | *p*-value | *r* | *p*-value | *r* | *p*-value | *r* | *p*-value |
| Anti-CCP | × | × | 0.09243 | 0.6749 | −0.14900 | 0.4975 | 0.41870 | 0.0468 |
| CRP | 0.09243 | 0.6749 | × | × | 0.02982 | 0.8925 | 0.11710 | 0.5947 |
| ESR | −0.14900 | 0.4975 | 0.02982 | 0.8925 | × | × | −0.07052 | 0.7492 |
| RF | 0.41870 | 0.0468 | 0.11710 | 0.5947 | −0.07052 | 0.7492 | × | × |

Additionally, the presence of anti-CCP antibodies in early disease is highly predictive for more rapid radiographic disease progression, a clinical hallmark of aggressive RA (Visser et al. 2002, Orbach et al. 2002, Vencovsky et al. 2003, Meyer et al. 2003, Forslind et al. 2004). Thus, Vencovsky et al. studied the predictive value of these autoantibodies in 64 patients with early RA. It has been found that anti-CCP positivity predicted progression of the Larsen score over two years better then RF (Vencovsky et al. 2003). These results were reinforced by Meyer et al. (2003) who evaluated sensitivity, specificity, positive and negative predictive value of anti-CCP in predicting radiological progression 5 years after observation. Recently, Forslind et al. (2004) reported in a prospective study that anti-CCP positive RA patients had significantly more joint damage than patients without this antibody. Prediction analysis showed that anti-CCP is an independent predictor of radiological damage and progression. Visser et al. (2002) described a clinical prediction model which includes evaluation of anti-CCP antibodies and can discriminate between self limiting persistent non-erosive and erosive arthritis. It have been claimed that anti- CCP antibodies were strongly associated with erosive arthritis more than RF (Odds ratio 4.56 vs. 2.99). Our previous study on 101 patients with RA clarified that anti-CCP is superior to RF as a predictive test for erosive RA (Orbach et al. 2002). The present study confirms previous suggestions that anti-CCP may reflect the development of joint damage in RA and anti-CCP is the best marker for erosive disease compared to other evaluated markers including MMP-3, RF, CRP and ESR. However, there are a substantial number of patients with this predictor, who still do not develop radiological damage in the near future. Thus, no single variable can assure correct diagnosis and prediction in an individual case, hence combined scores have been sought.

The value of anti-CCP and different RF isotopes for predicting the outcome of RA has been investigated recently. Vallbracht et al. (2004) evaluated anti-CCP antibodies and RF isotypes (IgM, IgA, IgG) in a large population of RA patients. They showed that IgM RF and anti-CCP are superior to other RF isotypes as a screening method for RA, and determination of both anti-CCP and RF isotopes contributes to the prediction of clinical disease activity and radiological damage. Additionally, up to 38.4% of IgM-RF negative sera exhibited reactivity against CCP. Therefore, in RF-negative patients detection of anti-CCP has convincing importance.

The additional diagnostic value of anti-CCP is even more impressive in the early course of RA, in patients with severe joint destruction and in patients with very active disease.

Thus, the study by Bus et al. (2003) in which the presence of IgM-RF, IgA-RF, and anti-CCP was evaluated, showed an association of IgA-RF and anti-CCP with clinical signs of disease severity. Similarly, analysis of RF isotopes (IgG-RF, IgA-RF, Ig M-RF) and anti-CCP antibodies in pre-disease serum samples revealed that anti-CCP and IgA-RF may predict the development of RA (Rantapaa-Dahlqvist et al. 2003).

### Conclusion

The results of our study suggest that the presence of anti-CCP antibodies has better diagnostic performance than MMP-3, RF, CRP and ESR in patients with erosive RA. We have demonstrated that this antibody is an independent predictor of radiological joint damage. Furthermore, anti-CCP antibody testing has been incorporated into newly proposed diagnostic criteria for RA and proved to be strongly associated with erosive disease. Further studies on larger patient populations are needed to assess the value of anti-CCP in clinical practice, especially for erosive RA.

### References

Bas S, Genevay S, Meyer O, Gabay C. 2003. Anti-cyclic citrullinated peptide antibodies, IgM and IgA rheumatoid factors in the diagnosis and prognosis of rheumatoid arthritis. Rheumatology (Oxford) 42(5):677–680.

Forslind K, Ahlmen M, Eberhardt K, Hafstrom I, Svensson B. 2004. BARFOT Study Group. Prediction of radiological outcome in early rheumatoid arthritis in clinical practice: Role of antibodies to citrullinated peptides (anti-CCP). Ann Rheum Dis 63(9):1090–1095.

Goldbach-Mansky R, Lee J, McCoy A, Hoxworth J, Yarboro C, Smolen JS, et al. 2000. Rheumatoid arthritis associated autoantibodies in patients with synovitis of recent onset. Arthritis Res 2(3):236–243.

Graudal N, Tarp U, Jurik AG, Galloe AM, Garred P, Milman N, et al. 2000. Inflammatory patterns in rheumatoid arthritis estimated by the number of swollen and tender joints, the erythrocyte sedimentation rate, and hemoglobin: Long term course and association to radiographic progression. J Rheumatol 27(1):47–57.

Keyszer G, Lambiri I, Nagel R, Keysser C, Keysser M, Gromnica-Ihle E, et al. 1999. Circulating levels of matrix metalloproteinases MMP-3 and MMP-1, tissue inhibitor of metalloproteinases 1 (TIMP-1), and MMP-1/TIMP-1 complex in rheumatic disease. Correlation with clinical activity of rheumatoid arthritis versus other surrogate markers. J Rheumatol 26(2):251–258.

Kirwan JR. 1997. The relationship between synovitis and erosions in rheumatoid arthritis. Br J Rheumatol 36(2):225–228.

Manicourt DH, Fujimoto N, Obata K, Thonar EJ. 1995. Levels of circulating collagenase, stromelysin-1, and tissue inhibitor of matrix metalloproteinases 1 in patients with rheumatoid arthritis. Relationship to serum levels of antigenic keratan sulfate and systemic parameters of inflammation. Arthritis Rheum 38(8):1031–1039.

Meyer O, Labarre C, Dougados M, Goupille P, Cantagrel A, Dubois A, et al. 2003. Anticitrullinated protein/peptide antibody assays in early rheumatoid arthritis for predicting five year radiographic damage. Ann Rheum Dis 62(2):120–126.

Nielen MM, van Schaardenburg D, Reesink HW, van de Stadt RJ, van der Horst-Bruinsma IE, et al. 2004. Specific autoantibodies precede the symptoms of rheumatoid arthritis: A study of serial measurements in blood donors. Arthritis Rheum 50(2):380–386.

Okada Y, Nagase H, Harris Jr, ED. 1987. Matrix metalloproteinases 1, 2, and 3 from rheumatoid synovial cells are sufficient to destroy joints. J Rheumatol 14:41–42.

Orbach H, Gilburd B, Brickman C, Gerli R, Shoenfeld Y. 2002. Anti-cyclic peptide antibodies as a diagnostic test for rheumatoid arthritis and predictor of an erosive disease. IMAJ 4:892–893.

Plant MJ, Williams AL, O'Sullivan MM, Lewis PA, Coles EC, Jessop JD. 200l. Relationship between time-integrated C-reactive protein levels and radiologic progression in patients with rheumatoid arthritis. Arthritis Rheum 43(7):1473–1477.

Posthumus MD, Limburg PC, Westra J, van Leeuwen MA, van Rijswijk MH. 2000. Serum matrix metalloproteinase 3 in early rheumatoid arthritis is correlated with disease activity and radiological progression. J Rheumatol 27(12):2761–2768.

Rantapaa-Dahlqvist S, de Jong BA, Berglin E, Hallmans G, Wadell G, Stenlund H, et al. 2003. Antibodies against cyclic citrullinated peptide and IgA rheumatoid factor predict the development of rheumatoid arthritis. Arthritis Rheum 48:2741–2749.

Schellekens GA, Visser H, de Jong BA, van den Hoogen FH, Hazes JM, Breedveld FC, et al. 2000. The diagnostic properties of rheumatoid arthritis antibodies recognizing a cyclic citrullinate peptide. Arthritis Rheum 43:155–163.

So A, Chamot AM, Peclat V, Gerster JC. 1999. Serum MMP-3 in rheumatoid arthritis: Correlation with systemic inflammation but not with erosive status. Rheumatology (Oxford) 38(5):407–410.

ACR Subcommittee on RA Guidelines. 2002. Guidelines for the management of rheumatoid arthritis. Arthritis Rheum 46:328–346.

Suzuki K, Sawada T, Murakami A, Matsui T, Tohma S, Nakazono K, et al. 2003. High diagnostic performance of ELISA detection of antibodies to citrullinated antigens in rheumatoid arthritis. Scand J Rheumatol 32(4):197–204.

Vallbracht I, Rieber J, Oppermann M, Forger F, Siebert U, Helmke K. 2004. Diagnostic and clinical value of anti-cyclic citrullinated peptide antibodies compared with rheumatoid factor isotypes in rheumatoid arthritis. Ann Rheum Dis 63(9):1079–1084.

Vencovsky J, Machacek S, Sedova L, Kafkova J, Gatterova J, Pesakova V, et al. 2003. Autoantibodies can be prognostic markers of an erosive disease in early rheumatoid arthritis. Ann Rheum Dis. 62(5):427–430.

Visser H, le Cessie S, Vos K, Breedveld FC, Hazes JM. 2002. How to diagnose rheumatoid arthritis early: A prediction model for persistent (erosive) arthritis. Arthritis Rheum 46(2):357–365.

Yoshihara Y, Obata K, Fujimoto N, Yamashita K, Hayakawa T, Shimmei M. 1999. Increased levels of stromelysin-1 and tissue inhibitor of metalloproteinases-1 in sera from patients with rheumatoid arthritis. Arthritis Rheum 38(7):969–975.

van den Berg WB. 2001. Uncoupling of inflammatory and destructive mechanisms in arthritis. Semin Arthritis Rheum 30(5 Suppl 2):7–16.

